# Oral contraceptives augment the exercise pressor reflex during isometric handgrip exercise

**DOI:** 10.14814/phy2.13629

**Published:** 2018-03-07

**Authors:** Clare Minahan, Hailey O'Neill, Nelie Sikkema, Sarah Joyce, Brianna Larsen, Surendran Sabapathy

**Affiliations:** ^1^ Griffith Sports Physiology and Performance Gold Coast Queensland Australia; ^2^ Menzies Health Institute Queensland Griffith University Gold Coast Queensland Australia; ^3^ Queensland Academy of Sport Centre of Excellence for Applied Sport Science Research Nathan Queensland Australia

**Keywords:** 17*β*‐estradiol, contraception, female sex hormones, metaboreflex

## Abstract

We sought to determine whether oral contraception alters the gender‐related differences observed in the exercise pressor reflex during isometric handgrip exercise. Fifteen men, fifteen normally menstruating women (WomenNM), and fifteen women taking monophasic oral contraceptives (WomenOC) completed two trials of a 3‐min isometric handgrip exercise protocol performed at 30% of their maximal voluntary contraction: (1) where arterial occlusion was applied to the previously exercising arm during a 3‐min recovery period (Occlusion trial); (2) where no arterial occlusion was applied during recovery (Control trial). Handgrip exercise elicited greater increases in mean arterial pressure (MAP) in MEN compared to both female groups (*P *<* *0.05), and in WomenOC compared to WomenNM in both trials (*P *=* *0.01, *P *=* *0.03). After 3 min of recovery, sBP was 12% (*P *=* *0.01) and 9% (*P *=* *0.02) higher in the Occlusion trial when compared to the Control trial for MEN and WomenOC. Conversely, arterial occlusion in recovery from handgrip did not sustain elevated sBP in the Occlusion trial, and sBP returned to recovery levels not different to the Control trial, in WomenNM (*P *=* *0.41). These data indicate that gender‐related differences in the metaboreflex during isometric handgrip exercise exist between men and normally menstruating women, but are blunted when men are compared to women taking oral contraceptives. We conclude that the suppression of 17*β*‐estradiol and/or progestogen in women via the administration of oral contraceptives attenuates sex‐related differences in the metaboreflex during isometric handgrip exercise.

## Introduction

The cardiovascular responses to exercise are regulated by a combination of efferent (central drive) and afferent (neural inputs from contracting skeletal muscle, arterial chemoreflexes, and baroreflexes) signals (Kaufman and Forster 1996; Nobrega et al. 2014). The reflex mechanisms originating in contracting skeletal muscles that adjust blood pressure (whilst also modulating heart rate and ventricular contractility) to meet the perfusive and metabolic requirements of exercise are collectively termed the exercise pressor reflex (EPR) (Nobrega et al. 2014). The EPR consists of the mechanoreflex that is sensitive to mechanical distortion, and the metaboreflex that is responsive to a range of metabolic by‐products produced by contracting skeletal muscles (Seals et al. 1988; Rowell and O'Leary 1990).

Men and women appear to rely on different physiological mechanisms to maintain cardiovascular control (Kneale et al. 2000; Reckelhoff 2001; Hart et al. 2009, 2011), and evidence suggests that the EPR is attenuated in women when compared to men (Ettinger et al. 1996; Jarvis et al. 2011). In a highly controlled study of men and naturally cycling women, Jarvis et al. (2011) demonstrated that women exhibited lower blood pressure (BP) and muscle sympathetic nerve activity (MSNA) responses compared to men during an isometric handgrip task performed at 40% of maximal voluntary contraction (MVC). Interestingly, when the contribution of the mechanoreflex was removed by ceasing handgrip, and arterial occlusion was applied in recovery, the disparate BP responses observed between men and women during handgrip remained, thus revealing a gender‐related difference in the metaboreflex (Jarvis et al. 2011). Based on the findings by Jarvis et al. (2011), and the previously reported cardiovascular protective properties of 17*β*‐estradiol (Mendelsohn and Karas 1999; Dubey and Jackson 2001; Rosano et al. 2007; Xue et al. 2009), it is reasonable to suggest that estradiol might account, in part, for gender‐related differences in the metaboreflex. Indeed, the cyclical fluctuation of female sex steroids across the menstrual cycle have been shown to alter the sympathetic control of circulation (Minson et al. 2000a,b; Charkoudian 2001). Even so, Jarvis et al. (2011) reported that higher (mid‐luteal) versus lower (early follicular) circulating sex hormone levels did not result in a further blunting of the BP and MSNA responses to isometric handgrip observed in women. These subsequent findings by Jarvis et al. (2011) and others (Petrofsky et al. 1976) raise some doubt in the notion that 17*β*‐estradiol (i.e., endogenous estrogen) is responsible for the gender‐related difference observed in the EPR. Nonetheless, circulating 17*β*‐estradiol concentrations remain elevated across the menstrual cycle when compared to men. Thus, long‐term exposure to 17*β*‐estradiol may act to blunt any inter‐phase difference in EPR while causing the observed gender‐related effect.

A comparison between normally menstruating women and women taking oral contraceptives provides an appealing experimental model with which to examine the effects of the long‐term suppression of 17*β*‐estradiol and/or the long‐term administration of ethinyl‐estradiol (i.e., synthetic estrogen delivered in the oral contraceptive pill) on the EPR. A comparison of the EPR among men, normally menstruating women, and women taking oral contraceptives would provide information regarding the gender‐related difference in the EPR as well as insight into the unique effects of 17*β*‐estradiol compared to exogenous estrogen (i.e., ethinyl‐estradiol) on cardiovascular physiology. In this study, we examined the BP and heart rate (HR) responses during isometric handgrip exercise with and without the application of arterial occlusion during recovery in both men and women. We hypothesized that the EPR response would be attenuated in normally menstruating women when compared to men. Furthermore, while the existing evidence is too scarce to formulate an evidence‐based hypothesis surrounding the effect of oral contraceptives on the EPR, it was tentatively predicted that the women taking oral contraceptives would have a similar BP and HR response to men due to the suppression of endogenous estrogen.

## Materials and Methods

### Ethical approval

The Griffith University Human Research Ethics Committee approved all procedures, and all participants provided written informed consent before commencing the study.

### Subjects

Fifteen men (MEN), fifteen normally menstruating women (WomenNM), and fifteen women who were taking oral contraceptives (WomenOC) volunteered to participate as subjects in this study. All subjects were recreationally active (moderate‐intensity, endurance‐type exercise 3–5 days/week for 30 min), nonsmokers, and did not have any documented history or clinical signs or symptoms of pulmonary, cardiovascular, or metabolic disorders. All WomenNM reported regular menstrual cycles (i.e., occurring on a 28 to 30‐days cycle) and had not taken any form of synthetic estrogen or progesterone for at least 6 months prior to the study. The WomenOC were using a combined monophasic oral contraceptive pill for at least 12 months (range 12–72 months) prior to the beginning of the study and continued their oral contraceptive pill throughout the experimental period. All subjects had never knowingly been pregnant.

### Experimental design

Subjects visited the laboratory on two separate occasions. The first visit was used to obtain written, informed consent, undertake preliminary health screening, and familiarize the subjects with the experimental procedures and equipment. All subjects were asked to complete a detailed medical history questionnaire that highlighted any illness or any other factor that may have prevented participation in the study. The investigator then explained all testing procedures and all related risks and benefits associated with the experiment before subjects were familiarized with the testing procedures and equipment.

During familiarization, subjects performed a maximal isometric voluntary contraction (MVC) using a hand dynamometer (Jamar, Sammons Preston, Bolingbrook, Illinois, USA). The grip span on the dynamometer lever was adjusted individually so that a comfortable grip was achieved. Subjects were instructed to squeeze the lever and exert maximal force for 3 sec with their right hand. Each subject was allowed three attempts and the highest of these were recorded as their MVC. Thirty percent of the MVC (30%MVC) was calculated and used as the workload for the experimental exercise protocol.

Subjects performed two, 3‐min bouts of isometric handgrip exercise during the second laboratory visit. Blood samples were collected before the start of the experiment during the early follicular phase of the menstrual cycle for WomenNM (i.e., day 2–6 of the menstrual cycle), and during the withdrawal phase for WomenOC (i.e., day 2–6 of placebo pill ingestion) for the subsequent analysis of serum 17*β*‐estradiol and progesterone concentration (Sullivan Nicolaides Pathology, Australia). MEN were tested at no specific time of the month. All exercise tests were conducted in the morning at least 2 h postprandial. Participants were instructed not to perform intense physical exercise or consume caffeine or alcoholic beverages for 24 h prior to each exercise test. All experimental testing was conducted in a climate controlled room to maintain temperature (24 ± 1°) and humidity (55 ± 2%).

### Isometric handgrip exercise protocol

The handgrip protocol consisted of two trials, both including 10 min of baseline rest, 3 min of isometric handgrip exercise, 3 min of recovery, and 5 min of rest. Each subject sat upright in a chair; the height of the chair was adjusted so that the shoulder was completely relaxed with no depression or elevation and there was 0° abduction of the upper arm. During handgrip exercise, the elbow joint was flexed at 90° and the forearm was supported on the table immediately in front in the anatomical position. During baseline, recovery, and resting measurements, subjects pronated their forearm to have their hand lie flat on the table facing palm down and relaxed.

After the 10 min baseline period, subjects were instructed to squeeze the lever of the handgrip dynamometer until the needle on the dial reached the predetermined load of 30%MVC. Visual feedback to the subjects and the investigators from the dial on the dynamometer ensured that 30%MVC was maintained for the full 3 min of the exercise period. Subjects were instructed to remain still during the 3‐min recovery and 5‐min rest periods and to leave their arm in the pronated position with their hand flat on the table. For one trial, arterial occlusion was applied to the previously exercising arm during the recovery period (i.e., Occlusion trial) whereas during the second trial, subjects returned their hand to the pronated position without arterial occlusion (i.e., Control trial). During the Occlusion trial, a sphygmomanometer cuff was placed on the upper (exercising) arm and inflated to a pressure of 230 mmHg immediately prior to the cessation of isometric handgrip exercise to obstruct blood flow during the entire 3‐min recovery period. The Control trial was identical to the Occlusion trial with the exception that the cuff was not inflated during the recovery period. The order of the two trials was randomized and separated by 25 min.

A CM5 electrode configuration with a Lohmeier electrocardiograph (M607, Munchen, Germany) was used to monitor cardiac rhythm and measure HR. Heart rate was recorded every minute during baseline (10 min) and at 15‐sec intervals during handgrip exercise (3 min), recovery (3 min), and rest (5 min). Averaged HR data from the final minute of each stage (baseline, exercise, recovery, and rest) are reported in the Results. A mercury sphygmomanometer (Standby model, W. A. Baumanometer Co Inc., Copiague, NY, USA) was used to measure BP. The sphygmomanometer cuff was placed on the subject's nonexercising (left) arm, at the level of the heart, and taped securely. A stethoscope was placed over the antecubital fossa and the cuff was inflated to ~180 mmHg. Systolic BP (sBP) was recorded at the onset of the first Korotkoff sound while diastolic BP (dBP) was recorded at the fourth Korotkoff sound. Blood pressure was determined by a trained Exercise Scientist with at least 3 yr experience in the measurement of blood pressure, and BP results are reported for the final minute of each stage.

### Statistical analyses

Subject characteristics were compared across the three groups using one‐way ANOVA. Fully factorial two‐way ANOVA with repeated measures for time was used to examine differences across trial (Control and Occlusion) and separately across group (MEN, WomenNM, and Women OC) for BP and HR. Pairwise comparisons with Bonferroni corrections were performed where a significant F value existed. Significance was accepted at *P *<* *0.05. All data are presented as mean ± standard deviation (for subject characteristics) or standard error (for all dependent variables).

## Results

### Subject characteristics

Subject characteristics are displayed in Table 1. MEN were significantly older than both groups of women (*P *<* *0.01), but there was no difference in age between WomenNM and WomenOC (*P *=* *0.47). Although WomenNM and WomenOC were of similar height (*P *=* *0.29) and body mass (*P *=* *0.06), MEN were taller (*P *<* *0.01) and heavier (*P *<* *0.01) than both the female groups. While there was no difference in progesterone concentrations between groups (*P *>* *0.05), MEN had significantly lower plasma estradiol concentrations when compared to WomenNM but significantly higher plasma estradiol concentrations than WomenOC (*P *<* *0.05). Similarly, WomenOC had significantly lower plasma estradiol concentrations than WomenNM (*P *<* *0.05). MEN demonstrated a higher MVC for handgrip (58 ± 2 kg) than both female groups (*P *<* *0.01), yet WomenNM (39 ± 2 kg) and WomenOC (39 ± 1 kg) demonstrated a similar MVC (*P *=* *0.79). The 30% MVC loads used by the MEN, WomenNM, and WomenOC were 17 ± 0.7, 12 ± 0.5, and 12 ± 0.4 kg, respectively.

**Table 1:** Subject characteristics of MEN, normally menstruating women (WomenNM) and women using oral contraception (WomenOC)

	Men (*n *=* *15)	WomenNM (*n *=* *15)	WomenOC (*n *=* *15)
Age (year)	25 ± 3^a^	21 ± 2	21 ± 2
Height (cm)	180 ± 9^a^	169 ± 7	166 ± 7
Body mass (kg)	75.9 ± 10.6^a^	65.7 ± 8.5	60.5 ± 5.5
Systolic BP (mmHg)	121 ± 9^a^	112 ± 6	110 ± 9
Diastolic BP (mmHg)	75 ± 10^a^	64 ± 7	68 ± 8
Plasma estradiol (pmol·L^−1^)	97.3 ± 23.0^a^	132.3 ± 41.7	45.9 ± 22.8^b^
Plasma Progesterone (pmol·L^−1^)	0.58 ± 0.12	0.78 ± 0.33	0.59 ± 0.11

Values represent mean ± standard deviation. MVC, Maximal voluntary contraction performed isometrically on a hand dynamometer with the right hand.

a Significantly different to both female groups.

b Significantly different to WomenNM; *P *≤* *0.05.

### Heart rate response during the isometric handgrip exercise protocol

**Figure 1:**
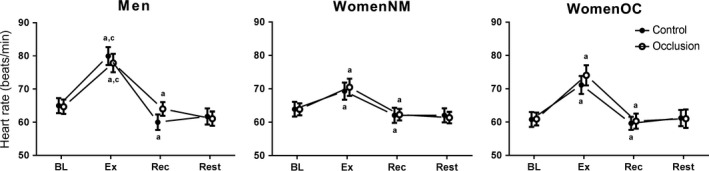
Heart rate measured in MEN, normally menstruating women (WomenNM) and women using oral contraception (WomenOC) at baseline (BL) and after 3 min (Ex) of isometric handgrip exercise performed at 30%MVC. Blood pressure was also measured after 3 min of recovery (REC) with (i.e., Occlusion trial = open markers) and without (Control trial = closed markers) arterial occlusion applied as well as after 5 min of rest (Rest) immediately following Rec without occlusion in both trials. (A) significantly different from previous time‐point within group and trial; (B) significantly different between from Control within group and time‐point; (C) significantly different from both female groups within trial and time‐point; (D) significantly different from WomenNM within trial and time‐point. Statistical significance accepted at *P *≤* *0.05.

Figure 1 presents the changes in HR measured before, during, and after isometric handgrip exercise in MEN, WomenNM, and WomenOC. Heart rate measured during baseline (HR_BL_) was not different among the three subject groups for either trial (*P *>* *0.05) or between the Occlusion and Control trials for any of the groups (*P *>* *0.05). Heart rate increased from baseline in MEN (Control trial = *P *<* *0.01; Occlusion Trial *P *<* *0.01), WomenNM (*P *<* *0.01, *P *<* *0.01), and WomenOC (*P *<* *0.01, *P *<* *0.01) after 3 min of 30%MVC handgrip exercise in both trials. The increase in HR during handgrip exercise (HR_EX_) was similar for both trials in all groups (*P *>* *0.05), but was greater in magnitude in MEN compared to both female groups (*P *<* *0.05). Heart rate during recovery (HR_REC_) was decreased from HR_EX_ in all groups during both trials (*P *<* *0.01), returning to values that were not significantly different from baseline and remaining stable during the 5 min of rest (i.e., HR_REST_) when no occlusion was applied in either trial (*P *>* *0.05).

### Blood pressure response during the isometric handgrip exercise protocol

**Figure 2:**
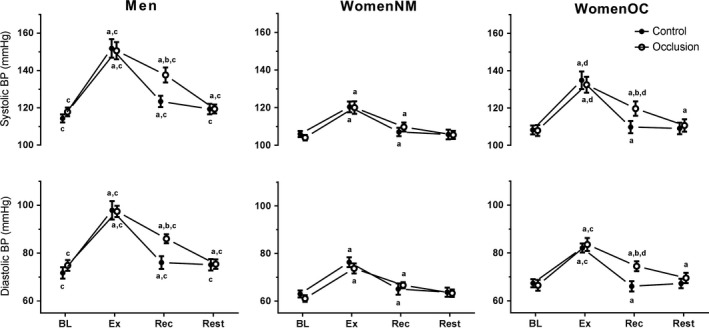
Systolic (upper plots) and diastolic (lower plots) blood pressure measured in MEN, normally menstruating women (WomenNM), and women using oral contraception (WomenOC) at baseline (BL) and after 3 min (Ex) of isometric handgrip exercise performed at 30%MVC. Blood pressure was also measured after 3 min of recovery (Rec) with (i.e., Occlusion trial = open markers) and without (Control trial = closed markers) arterial occlusion applied, as well as after 5 min of rest (Rest) immediately following Rec without occlusion in both trials. (A) significantly different from previous time‐point within group and trial; (B) significantly different from Control within group and time‐point; (C) significantly different from both female groups within trial and time‐point; (D) significantly different from WomenNM within trial and time‐point. Statistical significance accepted at *P *≤* *0.05.

Figure 2 illustrates the changes in sBP and dBP measured during the Occlusion and Control trials of isometric handgrip exercise. Baseline systolic (sBP_BL_) and diastolic (dBP_BL_) blood pressure were not different between the Control and Occlusion trials (*P *>* *0.05), but were higher in men compared to women in both trials (*P *<* *0.05). There were no differences in sBP_BL_ or dBP_BL_ between WomenNM and WomenOC in either trial (*P *>* *0.05). sBP and dBP increased from baseline during handgrip exercise in all groups (*P *<* *0.05). However, dBP_EX_ reached 98 ± 15 and 97 ± 9 mmHg in MEN during the Control and Occlusion trials, respectively; values that were higher (*P *<* *0.05) than that recorded for WomenNM (Control trial: 76 ± 8; Occlusion trial: 74 ± 8 mmHg) and WomenOC (Control trial: 82 ± 7; Occlusion trial: 84 ± 11 mmHg). Furthermore, dBP_EX_ was higher in WomenOC compared to WomenNM in both the Control (*P *=* *0.05) and Occlusion (*P *=* *0.01) trials. Similarly, sBP_EX_ reached 152 ± 19 and 151 ± 18 mmHg in MEN during the Control and Occlusion trials, respectively, and were higher (*P *<* *0.05) than the sBP_EX_ values for WomenNM (Control trial: 120 ± 11; Occlusion trial: 120 ± 13 mmHg) and WomenOC (Control trial: 135 ± 18; Occlusion trial: 135 ± 16 mmHg). sBP_EX_ was higher in WomenOC compared to WomenNM in both the Control (*P *=* *0.01) and Occlusion (*P *=* *0.03) trials.

After 3 min of recovery in the Control trial, dBP returned to values not different from dBP_BL_ in all groups (*P *>* *0.05), whereas sBP returned to values not different from sBP_BL_ in WomenNM (*P *=* *0.69) and WomenOC (*P *=* *0.62), but were still elevated in men (*P *=* *0.02). After 3 min of recovery in the Occlusion trial, dBP_REC_ was ~14% higher in both MEN (*P *=* *0.01) and WomenOC (*P *=* *0.01) when compared to dBP_REC_ recorded in the Control trial. In contrast, there was no difference in the dBP_REC_ measured in the Occlusion compared to the Control trial for WomenNM (*P *=* *0.57). Similarly, sBP_REC_ measured in the Occlusion trial were 12% (*P *=* *0.01) and 9% (*P *=* *0.02) higher for MEN and WomenOC when compared to the sBP_REC_ recoded in the Control trial. However, arterial occlusion in recovery did not alter the sBP response for WomenNM where sBP_REC_ was not different between the Occlusion and the Control trials (*P *=* *0.41). sBP and dBP returned to values not different between trials or from baseline values in MEN (Control trial, *P *=* *0.17; Occlusion trial, *P *=* *0.66) and WomenOC (Control trial, *P *=* *0.82; Occlusion trial, *P *=* *0.54) after 5 min of rest with no arterial occlusion in both trials.

## Discussion

We demonstrate here that, like men, BP measured in women taking oral contraception remains elevated above baseline when arterial occlusion is applied in recovery from handgrip exercise. This finding contrast that observed in normally menstruating women, where BP returns to baseline following isometric exercise despite arterial occlusion. In contrast, the heart rate responses across time and between experimental conditions were similar in men, normally menstruating women, and women taking oral contraception. This study provides evidence to suggest that oral contraceptive use attenuates the gender‐related differences observed in the EPR during isometric handgrip exercise, which has implications for the design of studies investigating the EPR utilizing female subjects.

Previous studies investigating the influence of gender on sympathetic nerve responses have reported higher peak HR as well as higher SBP and DBP values during isometric handgrip exercise in men compared to women (Ettinger et al. 1996; Wong et al. 2007; Jarvis et al. 2011). These findings may be explained, in part, by the greater work performed during 3 min of isometric handgrip exercise in men compared to women (MEN* *=* *22.9 ± 3.5; WomenNM* *=* *18.1 ± 2.5 kg/kg body mass; *P *<* *0.01), resulting in a greater activation of central command and/or afferent factors such as the EPR. In an attempt to control for the unequal “tension generated” between men and women, Ettinger et al. (1996) compared the muscle sympathetic nerve activity (MSNA) in men and women matched for MVC during thumb adduction exercise performed at 60%MVC. These authors reported greater MSNA in men compared to women, and suggested that the gender‐related differences in the sympathetic response to isometric exercise are independent of the inherent gender differences in active muscle mass. While we cannot rule out that the greater “total work” performed by men compared to women is responsible for the greater increase in BP and HR during exercise, the varying profile of the BP response during recovery from isometric handgrip exercise between MEN and WomenNM during the Occlusion trial suggests that the BP response may not simply be linearly related to work performed, particularly with respect to the contribution/influence of the metaboreflex.

Several authors have hypothesized that circulating 17*β*‐estradiol may be responsible for the gender‐related differences in BP responses to isometric exercise. Animal studies demonstrate that higher levels of estradiol are associated with enhanced lipid oxidation during exercise (Hatta et al. 1988; Kendrick and Ellis 1991; Ellis et al. 1994), minimising the interstitial concentrations of metabolic by‐products responsible for activating the metaboreflex (e.g., hydrogen ions, adenosine, and potassium). Alternatively, it has been suggested that 17*β*‐estradiol could increase blood flow to the muscle via endothelium‐dependent flow mediated dilation as a result of enhanced nitric oxide activity (Lieberman et al. 1994; Hernández et al. 2000; Khalil 2005). Improved blood flow could also reduce the activation of the metaboreflex by enhancing the washout of interstitial metabolic by‐products. Furthermore, an increase in the availability of nitric oxide as a result of high levels of circulating estradiol may increase *β*‐adrenergic‐mediated vasodilatation in the peripheral vasculature (Hart et al. 2011). By examining the BP responses in recovery from isometric handgrip exercise with arterial occlusion, we were able to isolate the metaboreflex from the mechanoreflex. BP measured during recovery from handgrip exercise during the Occlusion trial in this study was elevated above baseline in MEN, but not WomenNM, which is suggestive of a blunted metaboreflex in WomenNM. Our results support the findings of Ettinger et al. (1996) who found a reduced MSNA response in women during ischemic recovery from handgrip exercise compared to men, and lend support to the notion that chronically elevated 17*β*‐estradiol may play a role in reducing the activation of the metaboreflex during isometric exercise.

In order to further examine the proposed effects of 17*β*‐estradiol on the cardiovascular response to isometric exercise, we compared HR and BP values during and after isometric handgrip exercise between WomenNM and WomenOC. WomenOC demonstrated a greater increase in both sBP and dBP after 3 min of isometric handgrip exercise when compared to WomenNM. Given the two groups were matched for age, training status, and grip strength, and that the same total work was performed during the isometric handgrip exercise, it is unlikely that the greater increase in BP recorded during exercise for WomenOC was due to a greater activation of central command. Alternatively, we propose that the greater increase in BP measured during exercise in WomenOC compared to WomenNM was due to a greater activation of the metaboreflex. Indeed, sBP and dBP remained elevated above baseline values during recovery from isometric handgrip exercise during the Occlusion trial in WomenOC, but returned to values not statistically different from baseline in WomenNM. This suggests that, in contrast to WomenNM, the metaboreflex in WomenOC may be operating in a functionally similar pattern to MEN.

We demonstrated evidence of an attenuated metaboreflex in WomenNM during the follicular phase of the menstrual cycle when circulating concentrations of 17*β*‐estradiol are low. These findings, in agreement with Jarvis et al. (2011), suggest that acute variations at the concentrations of circulating 17*β*‐estradiol may not *directly* influence the metaboreflex. Rather, the long‐term suppression of 17*β*‐estradiol via the administration of oral contraceptives may result in alterations in genomic mechanisms responsible for gene and protein expression (Fischer et al. 2002; Kuhl 2005), that, in turn, influence the metaboreflex. Furthermore, research is warranted to explore the notion that long‐term suppression of 17*β*‐estradiol results in genomic alterations that may affect endothelium‐dependent vasodilation.

We cannot discount the effect of progesterone on the gender‐related differences in the EPR nor its effect on the EPR‐related differences observed between WomenNM and WomenOC. While no previous study has reported the independent effect of progestogen or progestin on the EPR, previous research demonstrates that progesterones influence cardiovascular regulation (Heesch and Rogers 1995; Brunt et al. 2013). For example, Brunt et al. (2013) report that endogenous progesterone (i.e., progestogen) blunts carotid vasomotor baroreflex sensitivity, while others show that progestogen modulates neurotransmitter release in medullary regions involved in autonomic regulation (Heesch and Rogers 1995). Therefore, further research is warranted to elucidate the independent effects of 17*β*‐estradiol/progestogen and ethinyl‐estradiol/progestin on the metaboreflex during isometric exercise. An additional limitation of this study is that, although all WomenOC in this study were taking a combined monophasic oral contraceptive pill, the exact type and dosage was not specified. It is possible that varying doses and types of progestins contained in monophasic oral contraceptive pills may have influenced its effect on the EPR (Africander et al. 2011). It is also possible that the subjects' pre‐exercise food consumption may have exerted a small influence on the measured variables, and thus, future studies should test participants in a fasted state. Furthermore, given a fixed‐duration protocol was adopted in order to directly compare the cardiovascular responses between groups at each time point, we cannot conclude what outcomes could have occurred had a test to fatigue been utilized. Finally, in order to provide a complete picture of the influence of OC on the metaboreflex, cardiovascular responses to isometric handgrip exercise with and without occlusion should be examined at various stages throughout the menstrual cycle and also across different forms of OC (i.e., biphasic, triphasic, progesterone only). Thus, future research should prioritize such investigations. Nevertheless, the current research provides important and novel insight into the effect of oral contraceptives on the EPR.

This study examined whether muscle metaboreflex control of BP and HR is influenced by differences in circulating estrogen and progesterone among men, normally menstruating women, and women taking the oral contraceptive pill. Interestingly, the increase in HR observed during isometric handgrip was not different between WomenNM and WomenOC, and the exercise‐induced increase in HR was not maintained in recovery from handgrip during the Occlusion trial in any group. This suggests that the metaboreflex does not act to adjust HR during isometric exercise. In contrast, we demonstrated that gender‐related differences in the metaboreflex control of BP during handgrip exercise exist between men and normally menstruating women, but are blunted when men are compared to women using oral contraception. We conclude that the suppression of 17*β*‐estradiol and/or progestogen in women via the administration of oral contraceptives attenuates sex‐related differences in the metaboreflex during isometric handgrip exercise.

## Conflict of Interest

The authors have no conflict of interests to declare.
